# The association of employment status and unwanted job loss with maternal oral health experiences: findings from the pregnancy risk assessment monitoring system

**DOI:** 10.1186/s12903-023-02869-4

**Published:** 2023-03-24

**Authors:** Alexander Testa, David Gimeno Ruiz de Porras

**Affiliations:** 1 Department of Management, Policy and Community Health, School of Public Health, The University of Texas Health Science Center at Houston, 7411 John Smith Drive, Suite 1100, San Antonio, TX 78229 USA; 2 Southwest Center for Occupational and Environmental Health, Department of Epidemiology, Human Genetics and Environmental Sciences, School of Public Health, The University of Texas Health Science Center at Houston, Houston, TX USA; 3 Center for Research in Occupational Health, Universitat Pompeu Fabra, Barcelona, Spain; 4 CIBER Epidemiología y Salud Pública, Madrid, Spain

**Keywords:** Oral health, Pregnancy, Employment status, Job loss, PRAMS

## Abstract

**Background:**

Oral health is an essential component of a healthy pregnancy. While most women work full-time while pregnant, research has overlooked the impact of occupational status and job loss on oral health experiences during pregnancy. To examine the impact of employment status and job loss on oral health experiences during pregnancy in the United States.

**Data:**

Data are from eight sites (Georgia, Massachusetts, Minnesota, Missouri, North Carolina, New York State, New York City, and Wisconsin) of the Pregnancy Risk Assessment Monitoring System (PRAMS) for the years 2016–2020 (*n* = 31,362). Multiple logistic regression is used to assess the relationship between occupational status (including employment status and unwanted job loss) during pregnancy and oral health.

**Findings:**

Women who experienced an unwanted job loss in the prenatal period were at elevated risk of not having dental insurance, not receiving a dental cleaning during pregnancy, having an oral health problem, and having unmet dental care needs.

**Conclusion:**

Experiencing unwanted job loss around the time of pregnancy is an important life event that corresponds to worse oral health experiences. There is a need for greater focus on adverse life events, such as job loss, especially during pregnancy, as a mechanism for oral health issues and challenges with proper access to dental health systems.

**Supplementary Information:**

The online version contains supplementary material available at 10.1186/s12903-023-02869-4.

## Introduction

Oral health is a critical component of overall health and well-being, [1] especially during sensitive periods of life, such as pregnancy. [2] Several features of pregnancy, including hormonal changes that contribute to inflammation, changes to dietary patterns, and nausea and vomiting that lead to tooth erosion, can contribute to increased risk for oral health conditions such as dental caries, gingivitis, and periodontal disease. [3, 4] Furthermore, oral health problems during pregnancy have been associated with various adverse pregnancy and birth outcomes, including preterm birth, low birth weight, and preeclampsia. [4, 5] However, estimates suggest that about half of women do not visit a dental provider during pregnancy, and nearly one-fifth of pregnant women report oral health problems. [6].

Many life circumstances may affect health-care-seeking behaviors, with employment being a primary example of such factors. Employment is beneficial for people’s health and well-being for the material (e.g., resources and access to affordable healthcare since employment in the U.S. is the main gate to health insurance) and psychosocial (e.g., support and peer networks practices) advantages it provides, which facilitate and foster self-care (e.g., such as regular visits for a dentist check-up). Indeed, in the United States, employment is a critical social determinant of health, [7] and persons with unemployment report worse mental and physical health and incur higher rates of stress-related illness. [8–11] In the U.S., most women work during pregnancy, [12] and increasingly, women who are employed work later into their pregnancy. [13] Importantly, unemployment and job loss around the time of pregnancy can contribute to hardship and pose challenges for maternal health. [14] While voluntary leaving a job is not uncommon, women who do so may be a selected group with enough resources and adequate access to health care. [12] By and large, though, unemployment and job loss, particularly when unwanted, can be highly detrimental when experienced around the time of pregnancy, as such events may lead to mental health problems, [15] feelings of loneliness, [16] and economic hardship, [14] which can negatively influence health behaviors and access to health care services. In the U.S., where lack of paid maternity leave is the norm, [17] these problems may be worse for women of low socioeconomic status who lack resources to buffer against economic contractions. [14].

Regarding oral health, experiencing unemployment or job loss during pregnancy can reduce the ability to afford dental services due to financial hardship and loss of dental insurance, weaken the individual’s networks, and thus reduce attention to self-care. While unemployment is a known risk factor for health [18] and poor oral health (i.e., dental caries, periodontal disease), [19, 20] limited research has investigated the connection between employment status, job loss, and oral health experiences during pregnancy. Extant research on the link between occupational status and oral health is scarce, and most of it is from outside the U.S. The small available literature suggests that most pregnant women receive insufficient dental care, [21–23] with the main barriers being financial stress (e.g., costs), [22, 24, 25] work-related aspects (e.g., time constraints, discrimination), [24–26] but also a lack of information about pregnant women’s oral health. [27] Employment status (e.g., related to financial and insurance access) and education (e.g., unawareness of oral hygiene or the effects of pregnancy on oral health) also have a role in the mother’s visit to dental care. [26, 28–30].

In summary, there is a need for more evidence to better understand the impact of unemployment and job loss experiences in the U.S., especially on one’s employment status in the prenatal period, on their oral health experiences, including connections to dental insurance, overall oral health, and access to dental care services. This study aims to fill this gap by examining the impact of employment status and unwanted job loss on maternal oral health experiences during pregnancy in the U.S.

## Methods

### Data

Data are from the Pregnancy Risk Assessment Monitoring System (PRAMS). The PRAMS are collected annually by the Centers for Disease Control and Prevention (CDC) and state health departments. Participating sites (states and territories) use birth certificate data to conduct a stratified sample of live births. The PRAMS data are collected from three sources: (1) birth certificate records, (2) vital record systems, and (3) a survey questionnaire. Surveys are sent to recent mothers through three mailing attempts 2-to-4 months following birth, and phone calls are made to non-responders within one week of the last mailed survey. Sites are included in the PRAMS only if they have met a minimum response threshold (55% from 2015 to 2017; 50% from 2018 to 2020). Because birth certificate records are available for responders and non-responders, as well as those not included in the PRAMS sampling frame, the sample is weighted to adjust for nonresponse and non-coverage. Thus, the PRAMS sample is representative of all live births in a given study site. Shulman et al. [31] provide additional information on the PRAMS study design and methodology. The CDC Institional Review Board approved using the PRAMS data for this study as part of the external researcher data-sharing agreement: https://www.cdc.gov/prams/prams-data/researchers.htm.

The PRAMS survey includes two components: (1) a core questionnaire sent to all study sites; (2) topic-specific questions asked of select sites in specific years. While the entire PRAMS covers approximately 48 sites (*n* = 206,080 live births from 2016 to 2020), representing 81% of live births in the United States, topic-specific questions are asked in a subset of sites. Specifically, between 2016 and 2020, 34 sites (*n* = 131,631) included questions on oral health experiences, 10 sites (*n* = 46,558) included questions on mother’s occupational status, and, 8 sites (*n* = 35,171) included questions on both mother’s oral health experiences and occupational status. After removing respondents with missing observations on main and control variables, the final analytic sample represents a pooled cross-section of 31,362 respondents (see Appendix A) who delivered a live birth in 2016–2020 in 8 sites in the U.S.

#### Outcomes

Several measures of oral health experiences during pregnancy were considered as outcomes. Consistent with prior research using PRAMS data, measures were dichotomized (yes = 0, no = 1) in the direction of expected risk (i.e., dental health problems). [32]*No dental insurance* was measured using responses to the statement: “I had insurance to cover dental care during my pregnancy.“ *Did not receive a dental cleaning* was measured by the statement, “during your most recent pregnancy, did you have your teeth cleaned by a dentist or dental hygienist.“ *Had a dental problem* was ascertained from the statement: “during the most recent pregnancy, I needed to see a dentist for a problem.“ *Received treatment for a dental problem* was measured with the statement: “during the most recent pregnancy, I went to see a dentist or dental clinic about a problem.“ Finally, *unmet dental care needs* were derived using the subset of respondents who reported they *needed to see a dentist for a problem* (n = 6,100) and a variable indicating whether or not the respondent *went to see a dental provider for a problem* (yes = 0; no = 1).

#### Exposure

Mother’s occupational status was derived using two dichotomous (0 = no, 1 = yes) PRAMS questions: (1) whether a respondent, at any point during her most recent pregnancy, worked at a job for pay and (2) whether a respondent reported having lost a job even though they wanted to go on working during the 12 months before the new baby was born. Using these two variables, four combinations were created: (a) employed and no unwanted job loss (reference category), (b) employed and unwanted job loss, (c) unemployed and no unwanted job loss, and (d) unemployed and unwanted job loss.

#### Covariates

To account for demographic, socioeconomic, and health insurance-related characteristics related to occupational status and oral health experiences, we included the following covariates: whether a *respondent’s husband/partner lost their job* in the 12 months before birth (0 = no, 1 = yes), the *mother’s age* (≤ 24, 25–29, 30–34, and 35 or older), *mother’s race* (White, Black, Asian, Mixed Race, or Other Race/Ethnicity), *mother’s ethnicity* (Not Hispanic, Hispanic), *mother’s educational attainment* (less than high school, high school graduate, some college, college graduate), *mother’s current marital status* (unmarried or married), *number of prior births* (0, 1, 2, or 3+), *number of dependents* (0, 1, 2, 3+), *household income* (≤ $16,000, $16,001-$40,000, $40,001- $85,000, > $85,000), *mother had no health insurance* in the month before pregnancy (0 = no, 1 = yes), *year of birth*, and *state of residence*. Finally, while having no dental insurance during pregnancy is an outcome variable, we use it as a covariate when assessing the receipt of dental care and oral health outcome variables.

### Statistical analysis

We first obtained estimates of unweighted counts and weighted prevalence of each variable. Then, we conducted bivariate analyses between oral health experiences and the mother’s occupational status. Then, variables with a *p*-value less than 0.25 in those analyses were entered into multiple logistic regression models for each outcome. Associations were expressed as the adjusted logistic odds ratio (OR) and 95% confidence interval (95% CI). Across models, variance inflation factors were below 2.5, indicating that multicollinearity was not an issue. [33].

Given that the impact of unemployment and involuntary job loss on oral health experiences may operate differentially across income levels (e.g., women with higher income levels may have resources to buffer against the impact of an involuntary job loss), we ran additional analyses for each oral health outcome stratified by income levels (Appendices B and C). All data analyses were conducted using the *svy* package for weighted survey data in Stata/S.E. version 17.

## Results

Weighted summary statistics are presented in Table 1. The most common group was composed of women who were employed and reported no unwanted job loss (66.7%), followed by 24.5% of women who were unemployed during their pregnancy and experienced no unwanted job loss during the 12 months before birth, 6.0% who were employed during their pregnancy and experienced an unwanted job loss 12 months before birth, and, finally, 2.8% who were unemployed during their pregnancy but had an unwanted job loss in the 12 months before birth. Across the oral health variables, 18.5% did not have dental insurance during pregnancy, 53.8% did not receive a dental cleaning, 18.5% had a dental problem, and 13.5% received dental treatment for a problem. Among the subsample with a dental problem, 33.8% had unmet dental care needs (i.e., reported having a dental problem, but did not receive dental treatment for a dental problem).

**Table 1:** Sample Characteristics (Unweighted N = 31,362; Weighted N = 2,397,133)

Characteristic	%	(*N*)
*Oral Health Experiences*		
Did not Have Dental Insurance	18.5%	(5,366)
Did Not Receive Dental Cleaning	53.8%	(17,096)
Had a Dental Problem	18.5%	(6,100)
Received Dental Treatment	13.5%	(4,581)
Unmet Dental Care Needs (*n* = 6,100)	33.8%	(2,007)
*Mother’s Occupational Status*		
Employed and no Unwanted Job Loss	66.7%	(20,663)
Employed and Unwanted Job Loss	6.0%	(2,176)
Unemployed and no Unwanted Job Loss	24.5%	(7,503)
Unemployed and Unwanted Job Loss	2.8%	(1,020)
*Husband/ Partner Lost Job*		
No	91.8%	(28,557)
Yes	8.2%	(2,805)
*Mother’s Age (in years)*		
< 24	19.4%	(5,744)
25–29	28.2%	(8,585)
30–34	31.7%	(10,203)
≥ 35	20.7%	(6,830)
*Mother’s Race*		
White	67.2%	(17,209)
Black	17.5%	(7,246)
Asian	6.3%	(2,903)
Other Race	6.1%	(2,205)
Mixed Race	2.9%	(1,799)
*Mother’ Ethnicity*		
Not Hispanic	84.9%	(26,409)
Hispanic	14.1%	(4,953)
*Mother’s Educational Attainment*		
Less than High School	9.7%	(2,998)
High School Graduate	22.1%	(6,690)
Some College	26.5%	(8,856)
College Graduate	41.7%	(12,818)
*Mother’s Currently Married*		
No	36.3%	(12,002)
Yes	63.7%	(19,360)
Number of Prior Births		
0	39.5%	(12,615)
1	33.6%	(10,047)
2	15.9%	(4,976)
3 or more	11.1%	(3,724)
*Number of Dependents*		
0	9.2%	(3,047)
1	34.2%	(10,883)
2	30.5%	(9,156)
3 or more	26.2%	(8,276)
*Household Income (in USD)*		
≤ 16,000	17.2%	(6,014)
16,000–40,000	22.8%	(7,250)
40,001–85,000	29.6%	(9,009)
> 85,000	30.4%	(9,089)
*No Health Insurance*		
No	87.4%	(28,059)
Yes	12.6%	(3,303)

**Fig. 1:**
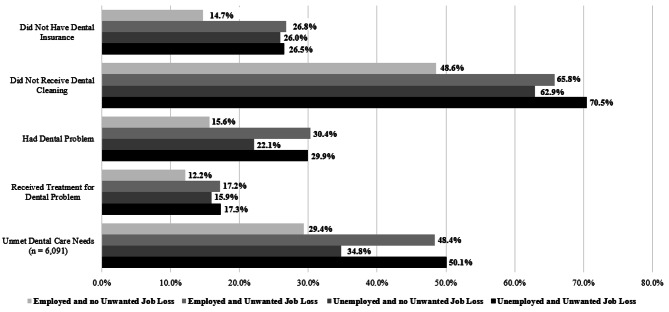
Prevelance of Oral Health Experiences by Mother’s Occupational Status

The general pattern (Fig. 1) shows that mothers who were employed and did not lose their job generally exhibited the best oral health experiences during pregnancy. In contrast, those who experienced an unwanted job loss 12 months before birth generally showed worse oral health experiences. Across all five outcomes, there were statistically significant associations (*p* < .001) between the mother’s employment status and oral health experiences during pregnancy.

In the multiple logistic regression models (Table 2), Model 1 showed higher odds of not having dental insurance among respondents who were employed and had an unwanted job loss (OR = 1.58, 95% CI = 1.33, 1.89), unemployed and did not have an unwanted job loss (OR = 1.36, 95% CI = 1.22, 1.51), and unemployed and had an unwanted job loss (OR = 1.31, 95% CI = 1.02, 1.69). The results in Model 2 similarly show that all three groups were associated with higher odds of not receiving a dental cleaning during pregnancy: employed and unwanted job loss (OR = 1.22, 95% CI = 1.06, 1.42), unemployed and no unwanted job loss (OR = 1.21, 95% CI = 1.11, 1.32), and unemployed and unwanted job loss (OR = 1.26, 95% CI = 1.02, 1.57).

**Table 2:** Association (OR, 95% Confidence Intervals) of Mother’s Occupational Status and Oral Health Experiences

	Model 1: Did Not Have Dental Insurance^a^		Model 2: Did Not Receive Dental Cleaning^a^		Model 3: Had Dental Problem^a^		Model 4: Received Treatment for Dental Problem^a^		Model 5: Unmet Dental Care Needs^b^	
Variables	OR	95% CI	OR	95% CI	OR	95% CI	OR	95% CI	OR	95% CI
Employed and no Unwanted Job Loss	—	—	—	—	—	—	—	—	—	—
Employed and Unwanted Job Loss	1.58***	(1.33–1.89)	1.22**	(1.06–1.42)	1.48***	(1.27–1.73)	1.08	(0.91–1.29)	1.77***	(1.36–2.31)
Unemployed and no Unwanted Job Loss	1.36***	(1.22–1.51)	1.21***	(1.11–1.32)	1.10	(0.99–1.23)	1.07	(0.95–1.21)	1.11	(0.91–1.35)
Unemployed and Unwanted Job Loss	1.31*	(1.02–1.69)	1.26*	(1.02–1.57)	1.38**	(1.10–1.72)	1.04	(0.81–1.34)	1.84**	(1.28–2.65)

***p < .001

Control variables include: mother’s Age, mother’s race, mother Hispanic, mother’s educational attainment, marital status, number of prior births, household income, number of dependents, no health insurance, husband/partner lost their job, state of residence, year of birth. Models 2–5 include a control variable for respondent did not have dental insurance.

^a^*n* = 31,362

^b^*n* = 6,100

Model 3 shows that respondents who experienced a job loss in the 12 months before birth exhibited higher odds of having a dental problem: employed and unwanted job loss (OR = 1.48, 95% CI = 1.27, 1.73); unemployed and unwanted job loss (OR = 1.38, 95% CI = 1.10, 1.72). In Model 4, we did not observe associations with receiving dental treatment for a problem. Model 5, which restricts the sample to the subset of respondents who reported having a dental problem (*n* = 6,100), shows that respondents who experienced a job loss in the 12 months before birth had higher odds of unmet dental care needs: employed and unwanted job loss (OR = 1.77, 95% CI = 1.36, 2.31); unemployed and unwanted job loss (OR = 1.84, 95% CI = 1.28, 2.65). Finally, analyses stratified by income levels (Appendices B and C) showed that oral health experiences were worse among women with lower incomes and improved as income levels increased across occupational statuses.

## Discussion

The findings showed a general pattern that suggests unwanted job loss in the prenatal period is associated with not having dental insurance, not having a dental cleaning during pregnancy, and having dental health problems. Notably, while unwanted job loss was associated with oral health problems, there was no association between employment status and unwanted job loss on receiving dental care for a problem. This finding raises the possibility that this group may have challenges accessing oral health care despite unwanted job loss being associated with elevated odds of oral health problems. Indeed, our analyses demonstrated that women who experienced an unwanted job loss had higher odds of experiencing unmet dental care needs. These findings highlight job loss as a critical but overlooked life event that corresponds with oral health problems during pregnancy.

The results of the current study point to several public health implications that can be beneficial in improving oral health experiences during pregnancy among women who have experienced job loss. First, one potentially helpful avenue is expanding public dental insurance options during pregnancy, especially after a job loss. Most adults have employer-sponsored dental insurance plans, and dental insurance remains an optional benefit in Medicaid, with no minimum standards. [34] This policy contributes to an unfortunate gap in dental care access considering that research finds expanding Medicaid adult dental benefits results in increased access to dental care. [35] Accordingly, extending dental insurance benefits to pregnant women, especially following a job loss through public programs such as Medicaid or The Special Supplemental Nutrition Program for Women, Infants, and Children (WIC), can be helpful to buffer against the shock of a job loss during this vulnerable period.

Second and relatedly, another consideration is altering the Affordable Care Act (ACA) to provide free or lost-cost dental care coverage to those who have lost a job—particularly during pregnancy—to better maintain low-cost connections to dental care services. Indeed, the American Dental Association has noted that while the ACA has made progress in expanding dental insurance, especially among children, the policy remains a missed opportunity to address access to dental care issues, particularly among low-income adults who face the most significant financial barriers to dental care. [36].

Third, while many women—especially those who experience a job loss—do not see a dental provider during pregnancy, prenatal visits can be an avenue to assess women’s oral health and create connections to dental care services. For instance, The American College of Obstetricians and Gynecologists suggests that “as part of routine counseling, health care providers should encourage all women to schedule a dental examination if it has been more than 6 months since their last examination or if they have any oral health problems.“ [37] This approach can be bolstered by training OB/GYN practitioners and primary care providers to screen for oral health problems, provide referrals to low-cost dental care services, and advise and educate patients on appropriate oral health hygiene practices.

Finally, a holistic approach, such as that offered by the Total Worker Health® framework, can guide efforts to improve the protections for working-age mothers to enhance their health and well-being. [38] Moving forward, it would be useful to integrate guidance for specific health issues, such as proper oral health practices and dental care access—especially for pregnant workers—into this framework. Such approaches can include the promotion of more oral hygiene practices in the workplace (i.e., providing floss, toothbrushes, and mouthwash), providing information on proper oral hygiene practices at work, enabling time for dental visits during work hours, and providing dental insurance plans that can help manage the cost of dental care for routine and emergency treatment.

### Limitations and future directions

There are limitations in the current study that can be addressed in future work. Given the cross-sectional nature of PRAMS, causality cannot be inferred. But our study nonetheless provides data to begin to fill in the gap on the impact of work or its absence or loss on oral health outcomes. The self-reported oral health measures in PRAMS could be subject to recall or social desirability bias. However, given PRAMS’ large sample size selected at random and the statistical weighting to control for nonresponse, such biases are likely to have a minor impact on our results. Further, reports of having an oral health problem were not confirmed with a diagnosis from a dental provider. Individuals with more severe problems might have a higher level of problem recognition than individuals with minor issues. If so, our findings will underestimate the actual magnitude of the association between occupational status and oral health problems. Relatedly, some variables, such as self-reported dental insurance during pregnancy, may be subject to measurement error as some respondents could be unaware of whether they have dental coverage. [39] In addition, the measurement includes oral health experiences during pregnancy, we cannot determine the onset of some conditions. For instance, the loss of dental insurance or the start of an oral health problem may have occurred before pregnancy for some women. Accordingly, the measurement of key variables related to oral health experiences, employment status, and unwanted job loss creates challenges in identifying the temporal nature of relationship. In the future, data collection efforts like PRAMS may need to introduce methodological changes to assess the timing of these events better.

Regarding the measurement of the occupational status, PRAMS does not include information on when precisely during the pregnancy the job loss occurred, the reason for the job loss, the duration of unemployment, or whether the mother received unemployment benefits that may have buffered against some of the economic shocks. Specifically, PRAMS’ question about unwanted job loss is asked to all eligible participants. Therefore, a “no” to this statement represents a heterogenous group, including mothers who did not lose a job in the prenatal period, mothers who did not have a job to lose, mothers who voluntarily quit, and mothers who were terminated from a job that they did not want to continue to work at. While the population prevalence of these different groups will vary greatly, we cannot assess their differential impact with PRAMS. Thus, future research, including more granular details on the status of employment conditions, is warranted.

Indeed, it is crucial for survey and epidemiological research to include questions related to occupational status and oral health measures in study designs, considering that the lack of data in this area makes researching questions about occupational status and oral health challenging. The measure of job loss in the current study pertained to involuntary job loss, which likely captures more at-risk individuals. However, it would be useful for future research to assess how certain forms of occupational status during pregnancy, such as voluntary job loss or moving from full-time to part-time, could influence oral health and may be protective by providing a pregnant individual with more time to engage in oral health care. Additionally, because questions on oral health experiences and occupational status are asked of a subset of states, the findings should not be generalized outside the eight locales included in the current study. Finally, while our study assesses how unwanted job loss is associated with oral health problems, reverse causality is also possible such that oral health problems contribute to job instability due to declines in work productivity because of oral health problems, [40] and the stigma of employers against those with oral health problems. [41].

## Conclusion

Our findings are novel since although prior research documents that oral health is essential for a healthy pregnancy, there is much less work examining the proximal risk factors around the time of pregnancy that corresponds to oral health experiences. Our study contributes to reducing this gap by bridging literature from occupational health and dentistry to assess how a woman’s unwanted job loss and employment status in the prenatal period corresponded with various oral health experiences during pregnancy. Our findings suggest that experiencing an unwanted job loss during pregnancy is an important life event that corresponds with higher odds of not having dental insurance, not receiving a dental cleaning, having a dental problem, and having unmet dental care needs. Therefore, there is a need for greater focus on adverse life events, such as unwanted job loss, especially during pregnancy, as a catalyst for oral health issues and challenges with proper access to dental health systems.

## Electronic supplementary material

Below is the link to the electronic supplementary material.

Supplementary Material 1

## Data Availability

The datasets generated and/or analyzed during the current study are not publicly available due to the nature of Pregnancy Risk Assessment Monitoring System not being publicly available. Data used in this study can be requested at https://www.cdc.gov/prams/index.htm. Queries about the data can be directed to Alexander Testa: alexander.testa@uth.tmc.edu.
