# Supplementary material for: The association of employment status and unwanted job loss with maternal oral health experiences: findings from the pregnancy risk assessment monitoring system
Source: BMC Oral Health. 2023 Mar 24;23:168. doi: 10.1186/s12903-023-02869-4 (PMC10039498; doi:10.1186/s12903-023-02869-4)
Supplement: Supplementary file 1 — Supplementary Material 1 [file 12903_2023_2869_MOESM1_ESM.docx]

| **Appendix A: List of States and Years in the PRAMS (2016-2020)** | | | | | | |
| --- | --- | --- | --- | --- | --- | --- |
| **State** | **Year of Birth** | | | | |  |
|  | **2016** | **2017** | **2018** | **2019** | **2020** | **Total** |
| Georgia | 0 | 731 | 603 | 626 | 573 | 2,533 |
| Massachusetts | 1,070 | 1,188 | 1,190 | 1,420 | 1,138 | 6,006 |
| Minnesota | 0 | 0 | 1,156 | 920 | 543 | 2,619 |
| Missouri | 949 | 979 | 832 | 1,423 | 818 | 5,001 |
| North Carolina | 0 | 871 | 802 | 756 | 0 | 2,429 |
| New York State | 807 | 741 | 654 | 663 | 0 | 2,865 |
| New York City | 1,013 | 902 | 1,051 | 893 | 789 | 4,648 |
| Wisconsin | 1,047 | 1,127 | 871 | 730 | 1,486 | 5,261 |
| **Total** | **4,886** | **6,539** | **7,159** | **7,431** | **5,347** | **31,362** |

| **Appendix B: Oral Health Experiences by Occupational Status and Income (N = 31,362)** | | | | | |
| --- | --- | --- | --- | --- | --- |
|  | **Employed and no Unwanted Job Loss (n = 20,663)** | | | | |
|  | **≤ $16,000**  **(n = 2,268)** | **$16,000 – $40,000**  **(n = 4,254)** | **$40,001 – $85,000**  **(n = 6,338)** | **> $85,000**  **(n = 7,803)** |  |
| *Oral Health Experiences* |  |  |  |  |  |
| Did not Have Dental Insurance | 22.2% | 20.2% | 17.7% | 7.6% |  |
| Did Not Receive Dental Cleaning | 65.6% | 64.2% | 52.8% | 33.1% |  |
| Had a Dental Problem | 29.1% | 21.5% | 15.6% | 9.3% |  |
| Received Dental Treatment | 21.4% | 15.7% | 11.4% | 8.6% |  |
|  | **Employed and Unwanted Job Loss (2,176)** | | | | |
|  | **≤ $16,000**  **(n = 821)** | **$16,000 – $40,000**  **(n = 682)** | **$40,001 – $85,000**  **(n = 476)** | **> $85,000**  **(n = 206)** |  |
| *Oral Health Experiences* |  |  |  |  |  |
| Did not Have Dental Insurance | 31.8% | 25.0% | 27.0% | 16.2% |  |
| Did Not Receive Dental Cleaning | 70.6% | 66.2% | 69.6% | 42.0% |  |
| Had a Dental Problem | 40.5% | 28.7% | 25.7% | 14.0% |  |
| Received Dental Treatment | 20.8% | 17.6% | 14.0% | 11.9% |  |
|  | **Unemployed and no Unwanted Job Loss (n = 7,053)** | | | | |
|  | **≤ $16,000**  **(n = 2,417)** | **$16,000 – $40,000**  **(n = 2,047)** | **$40,001 – $85,000**  **(n = 1,994)** | **> $85,000**  **(n = 1,043)** |  |
| *Oral Health Experiences* |  |  |  |  |  |
| Did not Have Dental Insurance | 25.1% | 30.5% | 26.5% | 17.7% |  |
| Did Not Receive Dental Cleaning | 71.4% | 66.7% | 60.5% | 42.6% |  |
| Had a Dental Problem | 24.2% | 24.6% | 22.3% | 12.2% |  |
| Received Dental Treatment | 16.4% | 17.9% | 16.2% | 10.8% |  |
|  | **Unemployed and Unwanted Job Loss (n = 1,020)** | | | | |
|  | **≤ $16,000**  **(n = 506)** | **$16,000 – $40,000**  **(n = 267)** | **$40,001 – $85,000**  **(n = 210)** | **> $85,000**  **(n = 37)** |  |
| *Oral Health Experiences* |  |  |  |  |  |
| Did not Have Dental Insurance | 32.0% | 17.9% | 25.0% | 22.4% |  |
| Did Not Receive Dental Cleaning | 75.7% | 69.3% | 63.5% | 52.3% |  |
| Had a Dental Problem | 32.5% | 28.9% | 27.6% | 14.1% |  |
| Received Dental Treatment | 17.4% | 20.5% | 14.7% | 7.4% |  |

| **Appendix C: Unmet Dental Care Needs by Occupational Status and Income (N = 6,100)** | | | | | |
| --- | --- | --- | --- | --- | --- |
|  | **Employed and not Unwanted Job Loss (n = 3,342)** | | | | |
|  | **≤ $16,000**  **(n = 643)** | **$16,000 – $40,000**  **(n = 968)** | **$40,001 – $85,000**  **(n = 989)** | **> $85,000**  **(n = 742)** |  |
| Unmet Dental Care Needs | 34.1% | 32.2% | 33.9% | 16.3% |  |
|  | **Employed and Unwanted Job Loss Lost Job (n = 707)** | | | | |
|  | **≤ $16,000**  **(n = 318)** | **$16,000 – $40,000**  **(n = 226)** | **$40,001 – $85,000**  **(n = 131)** | **> $85,000**  **(n = 32)** |  |
| Unmet Dental Care Needs | 51.0% | 48.4% | 50.9% | 16.1% |  |
|  | **Unemployed and not Unwanted Job Loss (n = 1,735)** | | | | |
|  | **≤ $16,000**  **(n = 665)** | **$16,000 – $40,000**  **(n = 506)** | **$40,001 – $85,000**  **(n = 431)** | **> $85,000**  **(n = 133)** |  |
| Unmet Dental Care Needs | 39.2% | 35.3% | 32.5% | 22.7% |  |
|  | **Unemployed and Unwanted Job Loss (n = 316)** | | | | |
|  | **≤ $16,000**  **(n = 183)** | **$16,000 – $40,000**  **(n = 76)** | **$40,001 – $85,000**  **(n = 50)** | **> $85,000**  **(n = 7)** |  |
| Unmet Dental Care Needs | 55.9% | 35.9% | 52.6% | 47.9% |  |
